# American Dental Association

**Published:** 1871-10

**Authors:** C. Stoddard Smith


### ARTICLE III.

### *American Dental Association.*

By C. Stoddard Smith, D. D. S.

The eleventh annual session of the American Dental Association was held in the Methodist Church, at Greenbrier, White Sulphur Springs, West Va., commencing on Tuesday, August 1st, 1871.

Soon after 10 o'clock the meeting was called to order by the President, Dr. W. H. Morgan, of Nashville, Tenn.

The roll of qualified members was called, and the reading of the minutes of the preceding meeting, was, on motion of Dr. G. H. Cushing, dispensed with.

The reports of standing committees were then called for, but, on motion of Dr. Crouse, the regular order was suspended in order to attend to some preliminary business. The hours of meeting were fixed at 10 A.M. and 3.30 P.M.

Inquiry being made in reference to clinics, the Committee of Arrangements reported that it would be difficult to hold clinics, as there were no operating chairs in the place.

Dr. Taft thought it not best to occupy valuable time in clinics. There will be none too much time to hear and consider the reports and other important topics, which ought long since to have been considered. The association could not afford to fritter away its time in manipulative exercises, which can be carried on in local societies. The influences of this body should extend all over the country, and further if possible. No clinics or exhibitions should be allowed during the hours for meeting.

It was finally decided that the clinics should be held from 8 to 10 A. M. The President gave notice that he should call to order punctually.

The regular order was then returned to, viz., the reading and consideration of reports of standing committees in consecutive order.

Dr. Cushing moved that Drs. Salmon and Morrison be, at their own request, discharged from service on the Committee on Instruments and Appliances, both having appliances of their own.

Carried.

Adjourned.

FIRST DAY—*Afternoon Session.*

After reading of the minutes, the Committee on Credentials asked for further time, which was granted. A partial report was, however, made.

The Committee on Dental Physiology was then called, and Prof. Judd, Chairman, submitted a report, of which the following is the substance:

Physiology is that branch of learning which treats of the phenemena of life. We say phenomena, because we know little of the essence of life; all speculations being too vague for our present purpose. Though physiology is the science of life, we most frequently mean by the word to include only function, without touching the question of life in the abstract. If, in this sense, we confine ourselves to a dental physiology, we have only to consider what is known in reference to the functions of the dental organs, which alone would present many fields for investigation. Whoever attempts to elucidate new principles in this field must first make himself master of the physiological literature of the day, or waste his time in experimenting on predetermined facts. If we study successfully the laws of life, we should direct attention to those conditions where its essental phenomena are exhibited in their simplest form, thus separating surroundings which might obstruct our view. Should we make our observations on the highest organized structures alone, we might conclude that an intelligent activity was an essential element of life; but if we go to the other end of the scale, we shall not make such a mistake. Life in the monad is as unmistakable as in the man. Nor is the vegetable world more simple. The veriest point of protoplasm exhibits all the essential characteristics of living beings.

Matter in its relation to life may be considered in four different forms. Heretofore, only three have been recognized, living, non-living, and dead, but we include one more viz: viable, and the category is complete. By viable matter we mean that which possesses only the potentialities of life; by living, that which possesses the activities; by non-living, that which is endowed with neither the activities nor potentialities of life; and by dead, that which has once lived but has ceased to manifest the activities, and no longer possesses the potentialities. There is a limit to our investigations which it were folly for man to attempt to pass. While the activities of life result from secondary causes, which may, and probably will, be understood, the philosophy of the potentialities is no more to be found out by scientists than that of chemical affinities, which would, if understood, involve a complete comprehension of Omnipotence. The potentiality of viable matter is vital, of non-viable,—as cohesion,—is non-vital. What answer can we give to the question, why does a cell possess the power of transforming the vital forces? and how is it that in their change common affinities are kept at bay? None, but to say that organic matter is the appointed medium of these changes. Such questions are beyond the scope of human intellect. When it is understood that different results obtained by the action of the same substances on living or non-living matter are determined by the different potentialities, the controversy as to the identity of forces will cease. We have no need to press into service unknown forces to account for the growth and development of a cell. The power of division is manifested when the mass—of cells—reaches a certain size. This power is the only essential process in the multiplication of living beings. But, although chemical investigations have led to astonishing results, nothing like an approach to the solution of the grand mystery of life has been achieved. Though man has read the storied pages of the world's primeval history, has discovered the laws of the evolution of central suns and planetary systems; though he has measured the depths of space and weighed its mighty orbs and determined the elements of which they are composed; though he has tamed the lightnings and made them his willing messengers, and thus anihilated time and space; yet, though every succeeding year may add to his knowledge, still the production of the simplest cell, with its powers of assimulation and division, will still be as far beyond his power as it is at present.

A word in relation to the innovation we have made by considering matter in four conditions may make evident the necessity for this new category. A seed entombed for thirty centuries with Egyptian mummies is still capable of germination; we state that during this period it has been *viable;* we do not assert that it is either dead or living, but that it is capable of life under favorable circumstances. But like dead matter, it must once have lived and ceased to exhibit the activities of life. We have, therefore, assigned it a special place in our category. The existence of a vital force, distinct from the physical, has never been demonstrated. The apparent necessity for such a distinction will disappear if we consider that different modes of motion are not different forces, but different manifestations of one force. Connect a piece of soft iron with the wires of a battery, the iron becomes a powerful magnet, though the connection made with other substances will produce no such results. The difference is owing to the different potentialities of the iron and the other substances. We have stated that the power of division and that of assimulation are the only distinct processes in the multiplication of beings. Though different methods of multiplication have been designated, as by division, by endogeneous growth, by a process of budding, or the pinching-off process, as it is termed, yet, if we observe these processes clearly, we shall find that they are essentially the same, simply divisions of the germinal mass. We do not refer to the antiquated doctrine of the formation of cells in a formative fluid. We are interested only in living tissues. We have confined ourselves to these few points, believing that the light shed upon them recently by the world's great luminaries warrants a modification of previous views.

Other papers on this subject being called for, Dr. Taft said that though it was the custom in local societies to read voluntary papers on any subject, yet such was not the case in this body.

Discussion on this subject being in order,

Dr. Atkinson said, regretting that he was not present when the first part of that report was read, he congratulated this body that a subject of this importance can be considered without that humdrum routinism which has borne down the medical profession. If he were disposed to speak with the mind of a critic, he might say that this is not a report on physiology but upon biology, which is the basis of life. We are inspirationally after truth. The present difficulty is the everlasting stickling for terms. He desired to get at the concept of the speaker, whether he was moved by his own inspiration or that of others. Matter has here been discussed in four forms. This is a new doctrine, but none the less truthful. The physicist must invade the field of consciousness, and know that he *can* think before he will know how to think. We have assumed a miserable duality instead of a glorious unity ; have divided matter and spirit, groping our way in darkness and despair, crying out that we shall never be able to know the truth. Knowing the beginnings, we will know the relations of things. You talk of a primal cell, and you don't know what a cell is. It ought to be spelled s-e-ll. Physiology means function, and that means organ. There is nothing that is non-living. Death itself is nothing but change. Everything comes under the law of homogeneous heterogenity and heterogeneous homogenity that pervades the universe. Going back to the beginning, I do not want to call these germs *cells*, but bodies. There are two ways of cognizing things,—by the senses and by the sentiency. All that comes in at the senses is physical; that which is cognized by the intellect—sentiency

—is immaterial. We come here to work on principles, on which our practice is based.

Dr. Taft offered a resolution inviting certain gentlemen, dentists and others, not members, to participate in the discussions, which, after considerable discussion was adopted.

Dr. Howe, of Cincinnati, said attention has been called to the confusion of terms in scientific discussions. Facts and truths are used as synonomous terms. Fact, however, is material; truth is revealed. There is an opposite to truth, but none to fact. Truth is found in revelation, and scientists will never be able to shake God's truth. Fact is matter in motion,—put in motion by spirit.

Prof. Judd desired to make an explanation. Dr. Atkinson believes that there is no essential difference between the crystal and the man, so far as vitality is concerned. I claim that vital actions are exhibited by living beings, and we call them vital because exhibited by living beings alone. When we say matter is dead, it is the language we have agreed to use when it fails to exhibit the phenomena of life. These are due to something which operates through dead matter. To understand this is beyond our comprehension. If Dr. Atkinson says that in the future we shall understand it, he can bring nothing forward to prove it. The word body I consider better than cell; but this word has been engrafted into our literature. Men know nothing about the growth of language; words apparently essential to the Latin tongue, and used by Cicero himself, have died out. Many words so change their signification as to mean exactly the opposite of what they once did. The French Academy attempted once to change certain words and substitute others, but found it utterly useless.

Dr. Atkinson. All that has been said has but proved true the inability of words to convey concepts. I will discuss philology in its proper place. I want to get men out of the place where I once was. Physiology is function,—that is, work of a body,—no matter whether we include it in the skin of a word or not. The concept of a cell boggles physiologists. Here is this great skin of ours, with its pabulum coursing within it; that is a great cell itself. The feeding,—that is physiology. Until the microscope was discovered we had not the concept of life. Hitherto shalt thou come and no further was written over the doors of physiology; but the glorious light of the Father above came streaming down through constructive lenses, and multiplied our conceptive powers. Anything that conspires to support functional activity is the basis of physiological action; anything that don't do this, arrests physiological action. It either supports or kills. Will Prof. Judd tell me what "killed" means?

Dr. Judd. Loss of its potentiality.

Dr. Atkinson. What do you mean by *its?*

Prof. Judd. Matter that has not only ceased to exhibit the actualities of life, but which has lost its potentialities.

Dr. Atkinson. We might spend a whole night in discussing whether a body frozen is dead or alive. If thawed slowly it resumes its functions, but if rapidly it becomes dead.

We have not yet risen to the conception that matter must be living to be at all. When patients come into our offices we assume wisdom, and say we understand it all; but until we do understand physiology we shall never understand its its difficulties. Plasma is chaos. Physiology is the taking in of plasmodic mass that feeds the bodies. Vitality is simply manifestation. We don't know what life or death is; it is change of habitat, of ghost. The law of togetherness and apartness is the most primal concept of life. Hence a crystal belongs as legitimately to the sphere of life as the seraph that blazes before the throne. The enamel of the dentine is the only substance that cannot be reproduced. All the rest may be. I know that bone and nerves may be reproduced; this can be demonstrated to stone-fence minds. The tissues cau be supplanted, as in the case of tuberculosis, where soft bodies are generated, taking the place of those by substitution. The case of Prof. Day, of Yale College, who was a pronounced consumptive at fifteen, illustrates this. He died at ninety-five, a few years since, having fallen in with a man who clearly saw that the pabulum must be supplied on which the tissues might feed. The tuberculosis became partly encysted and partly expelled, and the man recovered, when at thirty-five he was thought to be a hopeless case.

We have got to rise to a concept of the great truth that in God we live and move and have our being. Truth is revealed to us, and we must dispense it so that we may not look for things with the oblique rays that are only to be found out by the direct rays. When a thing is thus revealed we can speak with the certitude of the pillar of the throne of God. A crystal *does* think about what it is necessary to take in to perpetuate its own exisience. Here we have the key to the whole.

Prof. Judd. In relation to the whole matter, I would say that when Dr. Atkinson comes down to the facts, and I have seldom to call him to account as to facts, we do not disagree; but his inspirations and those of others lead in so many directions that I distrust them. I think there are times when a man sees things he cannot at other times; when he can speak as he could not at others; I will concede so much to his inspirations. I am not sure that we differ at all as to the facts in physiology. But when he comes to the upper strata, then I must leave him, and he ought not to quarrel with us if we don't believe in all his inspirations.

Dr. Atkinson. When our minds and affections are married we will not quarrel. We must integrate into our lives what the great Master taught, "He that will save his life shall lose it." If there is not a divinity in physiology, it is pathology.

The Chair announced the following appointments to fill vacancies on the various committees:

*Dental Physiology.*—Drs. R. Huey, of Philadelphia; and J. F, Thompson, of Virginia.

*Chemistry.*—Drs. H. A. Smith, of Cincinnati; and Webb and McDonneld, of Pennsylvania.

*Operative Dentistry.*—Drs. J. Taft, Cincinnati; W. Dutch, California; and C. D. Cook, New York.

*Mechanical Dentistry.*—Drs. W. N. Morrison, St. Louis; J. R. Walker, New Orleans; and E. Floyd, North Carolina.

*Dental Education.*—Drs. Francis, New York; Noble, of Washington; and F. Hickman, of Pennsylvania.

*Histology.*—Profs. Judd, and McQuillen.

*Dental Therapeutics.*—Drs. Atkinson, New York; J. J. Birge, of California; and G. W. Keely, of Ohio.

*Instruments and Appliances.*—Drs. J. F. Canine, Louisville; and Walker, of New Orleans.

*Prize Essays.*—Drs. Crouse, Chicago; J. I. Taylor, Cincinnat; and I. A. Salmon, Boston.

Dr. Taft suggested that though these committees could not be expected to prepare elaborate reports, yet they might touch upon a few points, so that definite action upon them might be taken.

Adjourned.

*To be continued.*

---

